# Soft touchless sensors and touchless sensing for soft robots

**DOI:** 10.3389/frobt.2024.1224216

**Published:** 2024-01-18

**Authors:** Chapa Sirithunge, Huijiang Wang, Fumiya Iida

**Affiliations:** Bio-Inspired Robotics Lab, Department of Engineering, University of Cambridge, Cambridge, United Kingdom

**Keywords:** touchless sensing, noncontact sensors, soft sensors, stretchable electronics, soft robots

## Abstract

Soft robots are characterized by their mechanical compliance, making them well-suited for various bio-inspired applications. However, the challenge of preserving their flexibility during deployment has necessitated using soft sensors which can enhance their mobility, energy efficiency, and spatial adaptability. Through emulating the structure, strategies, and working principles of human senses, soft robots can detect stimuli without direct contact with soft touchless sensors and tactile stimuli. This has resulted in noteworthy progress within the field of soft robotics. Nevertheless, soft, touchless sensors offer the advantage of non-invasive sensing and gripping without the drawbacks linked to physical contact. Consequently, the popularity of soft touchless sensors has grown in recent years, as they facilitate intuitive and safe interactions with humans, other robots, and the surrounding environment. This review explores the emerging confluence of touchless sensing and soft robotics, outlining a roadmap for deployable soft robots to achieve human-level dexterity.

## 1 Introduction

With recent emergence, bio-inspired soft robots gained the potential to possess extraordinary versatility and multifunctionality adopting biomimicry in many aspects (Mazzolai et al., 2022). Therefore, the design of such robots requires careful integration of sensing, passive and active mechanics, movement, and control (Ilami et al., 2021). These abilities can be accomplished through the integration of both soft and rigid materials into anatomies retaining global compliance and deformability (Coyle et al., 2018). Soft bodies have been used in combination with solid robots to achieve complex dynamics. Towards this end, exteroceptive sensing in soft robots which is underexplored at present has to be enhaced to realise smooth and humanlike capabilities (Pfeifer et al., 2012).

Soft robots are presumed to be more natural because of their pliability. Therefore, they are preferred for tasks that require physical contact (Jørgensen et al., 2022). However, soft robots can easily be deformed by external mechanical forces upon contact or stimuli and wear or deteriorate over reuse, causing them to fail faster than rigid-bodied robots. Tactile sensing, while providing excellent feedback to the environment, can accelerate the degradation process because of the potential for delimitation at the sensor-robot interface, which is known to be a weakness in soft robots (Johnson et al., 2021). Furthermore, the integration of encoders, strain gauges, and inertial measurement units hinders the flexibility of soft robots (Lee et al., 2017). Hence, the need for tactile sensing has certain limitations in the use of soft robots. Therefore, touchless sensing has emerged as a solution for such scenarios, thereby enhancing the sensing capabilities of soft robots with a faster and more efficient experience. This sensing mechanism is not intended to replace tactile sensing entirely, but to provide an opportunity for a soft surface to perceive its environment when wearing is an issue. However, touchless sensing has the potential to enable greater autonomy and closed-loop control in soft robots, particularly in practical and potentially hazardous environments, thereby enhancing safety (Thuruthel et al., 2018). Furthermore, adequate sensing of the surroundings provides a robot with proper sensory-motor self-organization by means of bio-inspired features.

In addition, the silicone structures used in soft robots require a minimum thickness to maintain structural integrity, which contributes to their overall size. The actuation mechanism employed in soft robots occupies a significant amount of space in their structure (Blumberg et al., 2013). To address these challenges and reduce the overall size and weight of soft robots, integration of touchless sensing alongside tactile sensing is crucial. Hence, soft robots can benefit immensely from touchless sensing technologies such as vision- or proximity-based sensors (Liu K. et al., 2022; Chen and Suo, 2022) to perceive and interact with their environment without any physical contact. By incorporating touchless sensing alongside tactile sensing, soft robots can to gather comprehensive information about their surroundings and make informed decisions, enabling safer and more efficient interactions in several applications (Lee et al., 2017).

The perception of the environment enables robots to effectively explore an unknown world and interact safely with humans and the environment. Among all extero- and proprioception modalities, the detection of mechanical cues is vital, as with living beings (Verrillo, 1992). In soft-bodied robots, the main difference centers on seamlessly combining actuation, sensing, motion transmission, mechanism elements and electronics must be combined into a continuum body that ideally holds the properties of programmable compliance and morphological computation (Alici, 2018). A variety of soft sensing technologies are currently available, but there remains a gap in effectively utilizing them in soft robots for practical applications, mainly due to their mechanical instability (Pal et al., 2021). This holds for soft, touchless sensing as well, which is a major branch of soft robotic sensing. Various constraints on soft robots to be mechanically perceived. For instance, there is no clear distinction between proprioception and tactile sensing in soft robots owing to their mechanical properties (Wang et al., 2018).

In this review, developments in soft robots with touchless sensing are summarized to provide a comprehensive understanding of the state-of-the-art in this field. Promising sensing technologies for touchless sensing for soft robots are described and categorized, and their advantages and disadvantages are discussed. Within this scope, we discussed touchless sensors which are made of compliant materials for use in soft robots and miniature touchless sensors which could be used in soft robots without causing damage to their structure or adversely impacting their flexibility. Strategies for designing touchless sensors and criteria for evaluating their performance are outlined from the perspective of soft robotic applications. Furthermore, the challenges and trends in the development of multimodal sensors, stretchable conductive materials and electronic interfaces, modeling techniques, and data interpretation for soft touchless sensing are highlighted. The knowledge gap and promising solutions for perceptive soft robots using touchless sensing are discussed and analyzed to provide a perspective in this field.

### 1.1 From human sensing to soft robotic sensing

#### 1.1.1 Tactile sensing

The human hand is covered with more than 17, 000 mechanoreceptors for sensing the surface pressure and vibrations (Vallbo and Johansson, 1984). These receptors can discriminate the shape, material stiffness, texture, and many other properties of objects and environmental phenomena. Hence, they represent a broad range of somatosensory capabilities. The skin around fingertips and palms also contains cutaneous receptors that can measure temperature, humidity, posture, and pain. Such multifunctional tactile sensing creates space for a wide variety of wearables (Pang et al., 2022) and human tasks, from grasping and dexterous manipulation (Song et al., 2022) to afferent touch and danger detection (Wang Y. et al., 2020). However, the accuracy and stability of movements, subsequently become unstable without touch (Westling and Johansson, 1984). Hence, human touch is more refined, sophisticated, dexterous, and often accompanied by emotional and social cues, such as comfort, intimacy, or aggression, than robotic touch. While robots are capable of sensing touch, they lack the complexity and versatility of human touch. The major obstacle is the technology is often not scaled up to complete systems in terms of multichannel, distributed, flexible, and resilient networks (Dahiya et al., 2009). However, new avenues have been explored to enhance the sensory capabilities of soft robots by incorporating human sensing principles into robotic systems, thereby providing them with an expanded range of perception. Soft-touchless sensors play a significant role in achieving this goal.

Multifunctional tactile sensors can mimic the sensing capability of human skin (Pang et al., 2022). Owing to the infinite degrees of freedom in soft robots, tactile sensing allows soft robots to access more information in the environment naturally and flexibly. However, this nature makes it difficult to define kinesthetics of a soft body accurately at the same time (Wang et al., 2018). Currently, grasping and manipulation are major applications of soft sensing. Tactile sensing allows soft robots to detect the shapes and textures of objects, which is crucial for grasping and manipulating objects effectively in unstructured environments. Tactile sensors closely interact with the environment compared with touchless sensors that interact with objects at a distance; hence, they can acquire more information. Among the multiple technologies behind touch sensing, capacitive and resistive technologies are the most prominent. In addition, sensors based on pressure (Kang et al., 2016) and infrared (IR) and acoustic waves have been developed. Highly deformable capacitive sensors are widely used for strain sensing in soft robots (White et al., 2017; Devaraj et al., 2019). Pressure localization on distributed sensory surfaces has been a priority in this type of sensing technology (Sonar et al., 2018).

While tactile sensing is essential in the field of soft robotics for dexterous tasks such as handling delicate objects, touchless sensing allows soft robots to interact with their environment without any form of physical input (Bartolozzi et al., 2016). A recent approach incorporating magnetic sensors that rely on embedded arrays of Hall effect sensors within an elastomer is shown in Hellebrekers et al. (2019). These sensors capture magnetic flux while adopting a touch-based sensing approach. However, the omnidirectional compliance of soft robots means that multiple sensors must be used to sense various modalities from different directions (Ang and Yeow, 2022). Hence, there is an interest in developing new techniques in which the characteristics of the materials used in sensors do not affect the intrinsic compliance of soft robotic components. Furthermore, touchless sensing is useful when no self-reference is required for perception, as in bio-inspired tactile sensors (Tanaka et al., 2011).

#### 1.1.2 Human vs. robotic touchless sensing

Human senses in-cooperate both tactile and touchless sensing where skin being the largest organ, and the eyes being the most powerful in terms of the amount of information acquired from the environment. Out of the five basic human senses, three are touchless; they are sight, smell, and hearing, with vision leading to sensorial impression (Rose, 2013). People without sight may compensate for that with enhanced hearing, taste, touch, and smell (Bauer et al., 2017). In humans, each sense uses a unique technology.

Eyes are the most powerful sense in humans in this regard. Sight or perceiving objects through the eyes, is a process where the light reflects from an object to the eye and multiple neural processes follow up until the brain perceives information out of it. The outermost, transparent layer of the eye, the cornea bends that light that passes through the pupil. Then the iris acts as the shutter of a camera, retracting to obstruct light and opening wider to allow more light (Roberts, 2016). The cogent confrontation between Physics and Biology has made the adoption of bio-inspired visual processes than any other sensing technology (Rose, 2013). What is worth observing here is the rich interconnectedness of human senses to other parts of the body. This holds for soft robots as well. If not one sense, another should compensate well for the robot to survive in challenging environments (Ang and Yeow, 2022). Therefore having touchless sensing gives the advantage of perceiving stimuli and challenges ahead, faster than touch. Nowadays, high-tech cameras, such as digital cameras or CCTV cameras, can have much higher resolution than the human eye. They can capture millions of pixels of information, whereas the resolution of the human eye is limited by the number of photoreceptors in the retina. On the other hand, high-tech cameras can be adjusted to operate in low-light conditions, and some cameras have specialized low-light sensors, which is not the case with the human eye. In addition, cameras have certain features such as sensitivity to a broader range of colors and wavelengths of the spectrum and a wide range of sizes and shapes that the human eye does not accommodate. However human vision is more complex in terms of variable resolution, rendering and processing power. Interestingly enough, the approximate limits to saturate human vision systems can be found in (Deering, 1998). For instance, the frame rate of the human eye is assumed to be equal to or above 60 Hz and very little is known about the interaction of rapidly varying complex rendered images in human vision.

Human sense of hearing, being mediated by the ears, detects sound waves, converts and amplifies them into neural signals (Roberts, 2016). In this regard, soft acoustic sensors and human ears both use sound waves to sense and interpret the environment, but they have some key differences. Soft acoustic sensors made of flexible and conformable materials, allow them to adapt to different shapes and environments (Gao et al., 2016). The human ear is highly sensitive to a wide range of frequencies, from 20 to 20,000 Hz, allowing us to hear a wide range of sounds from soft whispers to loud explosions. Furthermore, viscoelastic frequency-dependent dynamic properties of soft tissues in the human ear improve its directional sensitivity in addition to a range of sound intensities (Zhang and Gan, 2011). Soft acoustic sensors, on the other hand, are typically designed to detect specific frequencies or amplitudes of sound, and may not be as sensitive as human ears (Gao et al., 2016). The human ear can localize the source of a sound, and differentiate between speech and noise and hear sound while (soft) acoustic sensors typically have a narrower range of frequencies they can detect and typically require additional processing to interpret the signals they detect and are useful for specific applications, such as robotic grasping, navigation, and monitoring, while human ears are essential for overall survival, communication, and self-defense in the natural environment.

**FIGURE 1:**
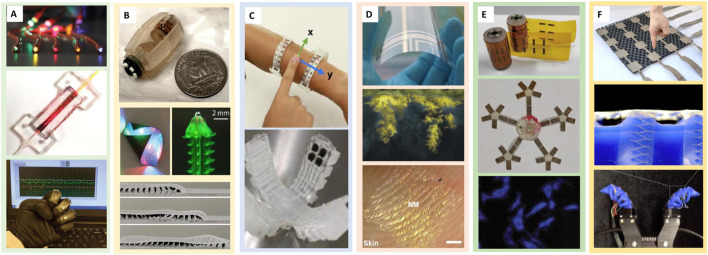
Overview of typical touchless sensors that have been applied in soft sensing. **(A)** Photosensitive soft sensors such as stretchable waveguide-based (Zhao et al., 2016) soft sensor (top), an optical soft sensor with fiber optic connectors (To et al., 2018) (mid), and photodiode-hydrogel hybrid-based spatial sensing (Chen and Suo, 2022) (bottom). **(B)** Magnetic-based sensing and the robot prototypes include the magnetic drug-delivery capsule (Kim and Zhao, 2022) (top), semi-fold electroplated serpentine circuits (mid-left) jellyfish-inspired swimming robot (Ren et al., 2019) (mid-right) and millipede-inspired crawling robot (Gu et al., 2020) (bottom). **(C)** IR sensor-based adaptive skin in soft interface and four-fingered gripper (Ogata et al., 2013). **(D)** Acoustic sensors such as coplanar antenna based on AgNPs sprayed to PET substrate (Bobinger et al., 2018) (top), hydrogel membrane implanted of silver dendrites (Gao et al., 2016) (mid) and hybrid MNs on the skin of a fingertip (Kang et al., 2018) (bottom). **(E)** Multi-leg robot with UV light sensing modules distributed onto each leg to achieve multidirectional UV light sensing (Dong et al., 2022) (top and mid). Fluorescence image of a cytochrome c (Cyt c) detection fluorescence sensor (GQDs-GO) (Cao et al., 2017). **(F)** Textile-only capacitive bimodal sensor array (Ye et al., 2022) (top) and multimodal sensor network integrated with a soft robotic gripper (Ham et al., 2022) (bottom).

Similarly, the sense of smell is mediated by receptors in the nose, which detect different chemicals in the air and send signals to the brain to create the sensation of smell. Odors mainly consist of hydrophobic volatile organic compounds with molecular weights of less than 300 Da and similar bioelectronic noses mimic smell using functional bioreceptors (Qin et al., 2023). They usually can be sensitive up to about 1–10 fM. The olfactory bulb in the brain processes the signals and allows us to recognize and differentiate various odors. This is similar to chemically reactive sensors in robots. All of these senses, including touch and taste, work together to create a rich and detailed experience of the world around us, allowing us humans to perceive and interact with our environment in a variety of ways. This is similar to multimodal sensors (Won et al., 2019). While taste traditionally involves direct contact with the tongue, taste is also considered somewhat touchless as it involves detecting soluble substances in food or liquids without the need for tactile sensing. However, taste typically involves contact but sometimes does not rely on the tactile sense to perceive flavors. Overall, soft sensors and human sensory organs have similar functions and requirements but differ in terms of materials, sophistication, sensitivity, processing, range, size, applications, and many other aspects. Mapping these objectives into morphology has been challenging and requires several stages of evaluation (Pinskier and Howard, 2022) and technology plays an important role in this process. In addition, it is worth noting that sensors have different technologies and principles of operation while human senses possess a single and unique mode of operation and slow evolution over time but are still more sophisticated. These aspects will be extensively discussed in later sections and several examples of soft touchless sensors are depicted in Figure 1.

In summary, human senses encompass a wide range of detection capabilities, such as vision and hearing; both of which can be categorised as touchless, while robotic sensors have specific operating ranges and principles of operation. Studying the sophistication of human touchless senses can provide an insight into the development of artificial soft, touchless sensors. However, due to the lack of technological and material advancements, soft touchless sensors have acquired a slow pace in being utilised in real-world applications and have comparatively less sophistication. Other than that, concerns related to robotic sensors in general, apply to soft touchless sensors as well.

### 1.2 Terminology

Touchless sensing is typically used to detect the presence, location, or motion of a body within the range of a sensor without any contact with the body. The terms proximity sensing, contactless sensing and touchless sensing have frequently been used interchangeably. However, touchless sensing covers a larger scope, including the sense of the presence of objects, distance to them, and discovering various properties associated with bodies without contact. Therefore touchless sensing is not limited to proximity sensing. By definition, touchless sensing refers to detecting or responding to physical or environmental changes, converting them into measurable signals that allow interaction or detection without the need for physical contact.

Although sensing has been discussed since the early stages of soft robotics, progress in touchless sensing has been slow compared to tactile sensing. The reasons for this slow progress are extensively discussed in Section 4. Both types of sensing: touch and touchless, together provide a robot with complementary information about the environment and objects, contributing to a more complete and accurate understanding of the surroundings. A major obstacle to using touchless sensing in soft robots is the lack of technology that can perform this task without affecting the viscoelastic properties of soft robots. Touchless sensing can be useful in prosthetics, wearables (Cianchetti et al., 2018) or anywhere soft and rigid materials are combined to perform a task. This eliminates the need for direct contact, reduces friction at the pressure points, and eliminates discomfort. It further promotes hygiene and cleanliness by avoiding the transfer of contaminants between the device and the environment, while providing flexibility and adaptability. This is a concern, as soft electronic devices with soft sensors have been developed for medical purposes (Zhang et al., 2020). For instance, touchless sensors can sense prosthetic limbs/joints that collide with each other, causing prosthetic devices to collapse when wearing them. Knee joints of a pair of prosthetic legs are examples of this. In addition, touch sensors have several inherent difficulties, including variations in the captured data owing to partial contact, wear, and tear owing to constant contact, nonlinear distortion, and inconsistent data quality To overcome these issues, touchless sensors can be used in parallel with touch sensors for the accurate operation of robotic components.

This review aims to provide a comprehensive overview of the current state-of-the-art soft touchless sensors, their significant recent progress, and bottlenecks in touchless sensors developed for soft robots, which address the problems for applications in the domain of robotic manipulation control and locomotion. Despite these improvements over decades, still, the challenge of developing capable soft robots with versatile touchless perception similar to rigid robots is still far from being resolved for real-world problems. Here, developments in soft robots with touchless sensing are summarized to provide a comprehensive understanding of the state-of-the-art in the field. Challenges and trends in touchless sensing, possible improvements to existing soft robotic touchless sensors, and directions for expansion are reviewed and discussed.

## 2 Touchless sensing technologies in soft robotics

The tactile sensing of robots has reached advanced technical development although not up to human level dexterity (Bartolozzi et al., 2016). Touchless sensing allows soft robots to interact with their environment without any physical input. Touchless sensing provides soft robots with several advantages, such as avoidance of the damage caused to the soft material by touch or repeated motion, being faster in acquiring information, and being ideal for compact environments.

Touchless sensors can be used in a wide range of applications including temperature measurement (Khatib et al., 2021), motion detection (Chen et al., 2021), inner body medical investigations (Wang J. et al., 2021), wearables (Runciman et al., 2019; Ying and Liu, 2021) and proximity sensing (Roberts et al., 2021). Owing to the contactless nature of these sensors, they are also used in situations where contact-based sensors are not practically viable or safe, such as in hazardous environments or with moving machinery. It is worth mentioning that the choice of touchless sensing technology depends on the specific requirements of the application and intended use. Additionally, some touchless sensing technologies have limitations, for example, IR sensors have difficulty sensing certain objects and media, whereas optical sensors can have problems in low-light conditions. The different types of touchless sensors are shown in Figure 1 and some common technologies behind these sensors are discussed in detail in later sections. For clarity, we categorise soft-touchless sensors based on the main sensing technique(s) behind their operation. However, currently most of these sensors have still been used for the detection of touch or touch-related features. This is because of the highly compliant nature of soft structures which resembles human skin. Hence these sensors have frequently been used for artificial skins.

### 2.1 Optical sensing and photosensitivity

Sensor morphology and sensory-motor coordination are important factors intertwined in determining the sensing capability of a device or a sensor. Inspired by insects, this is mainly because of the simplicity of these animals and ease of imitation. One aspect of sensor morphology is the distribution of the sensors (Brodbeck et al., 2015). In this regard, photosensitivity is an indirect means of tactile and visual sensing, which requires more than two sensor technologies. This increases the space and control requirements of robots. Photosensitive soft sensors respond to changes in light intensity or wavelength and can be used to detect the presence or absence of light, as well as changes in light intensity or color. These can be fabricated using various materials and techniques. One way to fabricate these is to embed photosensitive materials, such as photodiodes or phototransistors, into soft materials. These photosensitive materials can be integrated into soft materials using various methods, such as embedding, printing, or casting. Another method is the use of photoresponsive polymers, such as liquid crystals or photochromic dyes, which can change their properties in response to changes in light intensity or wavelength. These materials can be incorporated into soft materials using a variety of methods, such as casting, printing, or embedding. Additionally, some researchers have utilised bio-inspired photosensitive sensors, such as those found in the eyes of animals, to create soft sensors that can be used in soft robotics.

The optoelectronic skin is acknowledged as one of the world’s cutting-edge technologies in the fields of wearable healthcare monitoring, soft robotics, and artificial retinas (Liu K. et al., 2022). This adopts a surface energy-induced self-assembly methodology and is made of intrinsically stretchable phototransistors (ISTPTs), fabricated based on a stretchable photosensitive layer heterojunction with PQD films and hybrid polymer semiconductors. This extends to micro-scale arrangements on robots. Chen and Suo (2022) demonstrates a photosensitive soft skin consisting of optoionic sensors which behave as artificial nerves. A photodiode-hydrogel hybrid enables optoionic transduction, which is similar to the optical-to-ionic signal conversion in the human eye. This setup mimics a light-triggered reflex, such as blinking of the eye or camouflage of the skin.

A stretchable optoionic photodetector skin with very high resolution was presented in Liu K. et al. (2022). This sensor is capable of sensing both X-rays and UV light, with a variety of morphologies ranging from rod-like to worm-like morphologies based on the surface energy-induced self-assembly methodology. The worm-like CPQ QD film demonstrated a more uniform surface morphology and higher strain-tolerance capability than other CPQ QD films. Ultrahigh performance ISTPTs are heterojunctions consisting of worm-like CPB QD films and hybrid polymer semiconductors. ISTPTs fabricated using a stretchable photosensitive layer demonstrated highly sensitive responses to high-energy photons. The photosensing performance was better than that of the X-ray and UV photodetectors over time.

A deflection sensor is composed of a light source and light sensor, both of which are attached to a substrate, the subject of deformation in Dobrzynski et al. (2011). The sensor determines the angle of deflection by measuring the intensity of the light emitted from a light source and captured by the light sensor. This is based on contactless deflection measurements, where the softness of the substrate is unhindered as the deflection is performed using light. A miniaturised fibre optic gap sensor based on Fabry-Pérot interferometry was presented in Cosgun et al. (2022). This method can measure the absolute distance between close parallel surfaces, and the measurements are on the nanometer scale. This was originally used to monitor structural health; however, nowadays gap detection is also sought after in soft robotic applications.

Optical waveguide-based platforms are promising alternatives to classical electronics owing to their distinct advantages, such as EMI immunity, inherent electrical safety, and high stability in the long term wear or implantation. In addition to light transmission, waveguides can be designed and functionalized for highly sensitive sensing, such as strain, temperature, and bioanalytes. Waveguides made of typical materials are highly stiff and rigid, resulting in a significant mechanical mismatch between soft skin and tissues. To address these limitations, new types of waveguides made of soft, stretchable, biocompatible, and biodegradable materials have been intensively explored (Guo et al., 2019a). Currently, most stretchable fiber-optic sensors are intended to measure strain using implants or wearable devices (Guo et al., 2018; Guo et al., 2019b). However, these have the capacity to serve as carriers of small bits of information in addition to measuring pressure, stress, and strain. Implantable and biodegradable optical fibers for realizing deep brain fluorescence and optogenetic interrogation (Fu et al., 2018) and silk optical waveguides generated through direct ink writing waveguides Parker et al. (2009) can be considered a major step towards this.

Fiber optics are widely used in soft robotics for shape and elongation sensing by means of touch or embedment (Guo et al., 2019b; Galloway et al., 2019; Motwani et al., 2020; Yang et al., 2020). The favorable mechanical properties of fibre optics and the measurable distortion of light carried inside the optics are the reasons for this. However the requirement of a stronger light source on one end limits the use of fiber optics in soft robots. This is because they are flexible, durable, and can transmit signals over long distances with minimal losses. Although fiber optic sensors could not be an optimal choice for soft touchless sensing, sensing and fabrication technologies have made it challenging at present and Bai et al. (2020); Lepora (2021) testify to this. In addition, although soft cameras are not available, miniaturised cameras (Arbabi et al., 2016) can be helpful in maintaining the flexibility of soft materials as well as acquiring a large quantity of information compared to the rest of the sensors. The newly emerged soft and stretchable optical technologies will provide safe and reliable alternatives to next-generation, smart wearables and healthcare devices (Guo et al., 2019b). In addition to these soft sensors, rigid sensors have been used to take measurements of soft robots. This is possible as long as the sensors do not affect the compliance of the soft elastomers. For instance, an ambient light sensor was used to estimate the deformation of soft elastomers of different shapes (Sirithunge et al., 2023).

In summary, photosensitive touchless sensing has various applications, such as in wearables, e-skins, and as standalone sensors for robots or devices. Fabricating these sensors can benefit from using stretchable and biodegradable materials. In addition, stretchable fiber optics can be observed. The transmission, reflection, and refraction of light are commonly used to measure the light properties in these sensors. However, the progress of photo sensors has been limited to proximity sensors until recent years due to the complexity of handling light. In contrast, the abundance of natural light and its easy generation of light make it a widely researched area at present. Scattered light from surroundings is a major obstruction for developing sensory techniques based on natural light. In addition, sensors based on ambient light will not function properly in environments receiving limited light. This causes other types of radiation in the spectrum to be used in sensing. Because visible light is in the middle of the electromagnetic spectrum, photosensitivity remains one of the fastest sensing technologies in the field. Hence sensing sophistication of optical sensing and photosensitivity achieved a moderate speed over the past decade or so.

### 2.2 Capacitive sensing

Soft capacitive sensors can detect changes in capacitance caused by the proximity of an object or by changes in the physical state of the sensor and are typically composed of soft, flexible materials that can conform to the shape of an object and can be integrated into soft robotic devices.

A proximity sensor network fabricated by patterning two interdigitated comb electrodes is presented in Ham et al. (2022). As a conductive object approaches, a fringe field is generated between electrodes. However, these sensors can recognise an object’s proximity only up to a few millimetres. A dual responsive flexible iontronic skin that is capable of detecting pre-contact proximal events and tactile pressure levels is developed in Wang et al. (2023). Here, touchless sensing is attributed to the decrease in capacitance due to the inclusion of objects in the fringing electric fields. For tactile sensing, the sensor uses capacitance variations of the iontronic skin originating from the dimensions and contact area of an object under pressure. Proximity sensing uses the change in capacitance originated from the disturbances of the inserted fringing electric field. The capacitance-based touchless sensing was used for material categorisation between polymer, metal, and human skin at a distance of 2 mm from the object.

Alshawabkeh et al. (2023) is an example of a stretchable capacitive sensor for proximity and tactile sensing for a soft robotic finger. Proximity sensing recognises five different materials such as aluminium and wood, based on their electrical permittivity values, and the sensor was fabricated using a 4-layer electrode structure. This was sensitive to a distance of approximately 25 mm during the testing, depending on the material.

Capacitive sensors have been reported to improve the permeability of flexible capacitive sensors due to their inherent nature of lightweight, breathability, discretion, deformability, softness, and comfort, and soft capacitive sensors in wearable testify to this (Dobrzynska and Gijs, 2012; Mishra et al., 2021). To improve the flexibility and wearability of capacitive sensors, polymer elastomers, including polydimethylsiloxane (PDMS), polyethylene terephthalate (PET), polyimide (PI), polyvinyl alcohol (PVA), silicone (siloxane compounds), and polyvinylidene fluoride (PVDF), are often used to prepare flexible electrodes and dielectric layers. While most of these are suitable for contact sensing, capacitive sensors have the ability to act as both contact and noncontact sensors. Hence they have been widely applied owingto their outstanding temperature insensitivity, low power consumption, rapid dynamic response, and simple architecture design (Ye et al., 2022). The ease of fabrication, higher scalability, and ruggedness makes capacitive sensing one of the most suitable technologies for integrating into soft structures when sensing principles are considered (Alshawabkeh et al., 2023).

To fabricate a soft capacitive sensor, a layer of conductive material, such as metal or carbon nanotubes, is deposited onto a flexible substrate, such as polymer or rubber. The conductive layer is then insulated from the substrate using a nonconductive layer, such as a dielectric. The resulting sensor can detect changes in capacitance by measuring changes in voltage across the conductive layer. This is caused by the proximity of an object or changes in the physical state of the sensor.

Capacitance has been used in both tactile and touchless sensing. Capacitive sensors are widely used in soft robotics for proximity and touch sensing. They can be used to detect the presence of an object, measure its distance, and detect changes in pressure or force applied to a sensor. They are also useful for sensing positions and deformations of soft robotic devices. Soft capacitive sensors are relatively simple to fabricate, robust, and can be integrated into a wide range of soft robotic applications. The nonlinearity of output and sensitivity to environmental changes are major concerns in using capacitive sensors. Currently, new materials which possess better responses for noise and environment conditions are being utilised in the fabrication of capacitive sensors to overcome this issue. Capacitive sensors achieved a moderate sensor sophistication due to the advancement of materials and fabrication techniques over the last few decades.

### 2.3 Magnetic sensing

Soft magnetic sensors can detect changes in magnetic fields caused by the proximity of an object or changes in the physical state of the sensor. Magnetic soft robots can not only work in the biomedical field inside the human body using micro-scale design concepts, but also have applications in industrial production processes, logistics, medical operations, and automotive fields (Wang et al., 2022b). To sense mechanical changes and convert them into electromagnetic signals, thus enabling sensors based on the magneto-control principle. Developments in fields such as robotics require an increased ability to sense mechanical stimuli in the environment, such as touch, vibration, and fluid sensing, and the stress response that occurs in magnetic stimulus-responsive polymers in a magnetic field allows smart composites with the ability to be used in developing sensors (Cai et al., 2018).

The soft magnetic microelectromechanical (m-MEMS) skin developed by Ge et al. (2019) enables the interplay with physical objects enhanced. Both tactile and touchless interactions are enabled simultaneously in a single compliant wearable sensor platform. The sensor is encapsulated with a polymeric foil hosting an array of four Giant Magneto Resistance (GMR) sensors which are actuated upon the presence of a magnetically functionalized object for touchless interaction and by mechanical deformation of the m-MEMS package upon application of pressure for tactile interaction. The sensor can specify the magnetic objects out of the irrelevant nonmagnetic objects with signal-programmable manipulation of the objects by adjusting the magnetic properties of objects of interest. This resembles the natural skin which not only readily distinguishes different types of stimuli but is also sensitive over a wide range of signal intensities (Edwards and Marks, 1995).

Functional soft materials have recently emerged with the capability to sense external stimuli, such as heat, light, solvent, or electric or magnetic fields. Origami and capsule robots have pioneered this study Kim and Zhao (2022). Multimaterial 3D printing facilitates the integration of sensing and actuating components, where additional sensing elements should not affect the deformability of the platform. Hence, highly compliant, stretchable conductive polymers or hydrogels based on poly (3,4-ethylenedioxythiophene)-poly (styrene sulfonate) have been used in such applications Yang (2022); Stottlemire et al. (2021).

Elastic solids with mechanical softness or compliance, as well as magnetic properties which are called ‘magnetic soft materials’. Additive manufacturing techniques, microfabrication, micromolding, and microassembly have accelerated the application of such materials, including small-scale untethered soft robots and flexible electronics (Kim and Zhao, 2022). Magnetic soft robots are more commonly used than magnetic sensors in soft robots (Dong et al., 2022). A m-MEMS platform is realized by packaging a flexible magnetic field sensor and a compliant permanent magnet with a pyramid-shaped extrusion at its top surface into a single architecture in Ge et al. (2019). The lack of fabrication techniques, design, and compatible materials for such sensors is the main reason for this. In addition, magnetic actuation has achieved more progress in recent years than has magnetic sensing. Ultimately, magnetic microelectromechanical systems enable complex interplay between physical objects and robots. This interaction is enriched with virtual data and used in many areas including augmented reality, robotics, and medical applications.

To fabricate a soft magnetic sensor, a layer of magnetic material, such as a ferromagnetic or superconductive material, is deposited onto a flexible substrate, such as a polymer or rubber. The magnetic layer was insulated from the substrate using a non-conductive layer. In addition, ferromagnetic fluids, which can be embedded into soft materials, have become popular among researchers of soft sensing technologies. The resulting sensor can detect changes in the magnetic field by measuring changes in the voltage or resistance across the magnetic layer caused by the proximity of an object or by changes in the physical state of the sensor.

In summary, magnetic soft touchless sensors are widely used in soft robotics for proximity and position sensing. They can be used to detect the presence of magnetic objects, measure the distance, and detect changes in magnetic field strength. They can also be used to sense positions and deformations of soft robotic devices. These possess high sensitivity and can sense magnetic fields in environments with high levels of noise or interference. However the high levels of interference from certain magnetic fields can disturb the sensor as well. Therefore applications of magnetic sensors can be limited depending on the environment in which they are used. However, recent progress in soft magnetic materials and fabrication techniques specific to magnetic sensors leveraged their development. Due to these reasons, a moderate sophistication has been achieved for magnetic sensing over the past decade.

### 2.4 Infrared sensing

Infrared sensing can be useful for a variety of applications, such as proximity and visual sensing, where an IR sensor can detect IR light levels and can be used to navigate or locate objects in IR-rich environments. Detecting temperature changes is one of the main applications of this type of sensors (Yamaguchi et al., 2019).

An IR-based MEMS proximity sensor for autonomous vehicles was simulated in Muthuviswadharani et al. (2016). An improved noise output was obtained through simulations. SenSkin is an IR-based photosensitive array in the form of an armband that measures skin deformation (Ogata et al., 2013). This is an example in which both soft and rigid materials have been combined to create a wearable partly soft sensor. A telescopic, pneumatic, soft palm was developed in Meng et al. (2020) to avoid damage to an IR distance sensor caused by a potential collision. High-performance silicone rubber, hybrid deposition manufacturing (HDM) technique, and multistage molding have been used for the fabrication of this platform.

To sum up, IR photodiodes (Wang C. et al., 2020) and IR sensors such as pyroelectric sensors (Dao et al., 2019), and IR proximity sensors (Kito et al., 2019) have commonly been used for IR detection. IR LEDs are commonly used as IR light sources on such occasions. During the fabrication process, IR sensing should be integrated with soft fabricates as in other soft sensors. The availability of soft IR touchless sensors is currently limited because of the challenge of identifying stretchable materials that are compatible with IR sensing. Consequently, an alternative solution is to explore interfaces that can combine soft and rigid sensory platforms without harming them. IR sensors achieved low sensor sophistication and slow progress due to the above reasons over the last few decades.

### 2.5 Ultaviolet sensing

Ultraviolet (UV) sensing in soft robotics can be useful for a variety of applications such as detecting UV light sources or changes in UV exposure. UV sensing can also be used in soft robotics for visual sensing, where a UV sensor can detect changes in UV light levels and navigate or locate objects in UV-rich environments.

The fluorescence nanosensor based on graphene quantum dots (GQDs) supported by graphene oxide (GO) in Cao et al. (2017), is an example for UV-based sensing and hence ideal for fluorescence “turn-on” and “turn-off” and nanoscale biological imaging. Hence this can be ideal for many soft robotic inner-body applications. The multifunctional soft robot in Dong et al. (2022), has UV light and temperature sensing particles integrated into soft robots where they quickly change their color from white to green in the presence of UV light. Flexible UV sensors are popular in wearables and there are various approaches for fabricating flexible *ZnO*-based UV sensors on different substrates such as solvent-free direct drawing of *ZnO* on a cellulose paper by a *ZnO* pencil, screen-printed *ZnO* nanowires, inkjet printed *ZnO* nanowires and selective laser writing (Zou et al., 2020). The detectivity and conformability change according to the fabrication techniques and materials associated with each technique. *ZnO*, *TiO*_2_ -based systems, such as thin films and nanotube arrays, have also been well-studied and have well-established fabrication strategies for morphological control such as thin films, nanotube arrays etc. Traditional semiconductors such as *ZnO* and *TiO*_2_, *CNTs* have also shown significant promise in the development of (photoelectric) UV sensors. They possess distinct characteristics such as lightweight, high surface area, high electrical and thermal conductivity, high mechanical strength, high flexibility, and excellent stability which explore the path towards widespread use in sensing applications.

A millimeter-scale, flexible, wireless dosimeter operating across a wide range of the electromagnetic spectrum (UV to IR) was introduced in Heo et al. (2018). The fabrication of the device includes a simple set of procedures using a laser structuring tool, galvanic pulsed electroplating system, and a pick-and-place machine. The substrate is a thin, flexible sheet of polyimide clad in rolled and annealed copper. A transparent coating of poly (dimethylsiloxane) (PDMS) or UV-transparent optical adhesive Norland Optical Adhesive (NOA) was spontaneously applied to the entire device in liquid form forming into a smooth curved shape through the action of surface tension. This step is followed by thermal or photo curing which yields a solid material that makes active components waterproof.

There are several reasons for the limited use of UV-based touchless sensors. The main reason for this is that the safety concerns regarding UV are higher. UV light can be harmful to human skin and eyes if proper precautions are not taken (D’Orazio et al., 2013). The deployment of touchless sensors using UV technology requires careful consideration of safety measures to protect individuals from potential harm. Another reason is that alternative touchless sensing technologies exist. For example, natural light is readily available in surroundings and is easily generated from artificial sources. Therefore, it can replace UV technology for soft sensing. Similar sensing technologies are capacitive and IR which are lower cost, reliable, and easy to integrate compared to UV sensors. Therefore the complexity and cost of implementing such systems may increase. In addition, UV light can be obstructed by physical barriers, which affect the functionality of the sensor. Another reason is the lack of research on UV technologies for sensing and soft robotics in general. These limitations can slow the development and deployment of UV sensing and may make other touchless technologies more suitable for specific applications. Over the last few decades, wearable UV sensors have achieved greater progress over the last few decades in terms of salient technologies, that is, photoelectric and photochromic UV sensors (Zou et al., 2020). Significant challenges in the design principles of such technologies include the requirements for high performance, cost-effectiveness, and the production of user-friendly flexible devices.

The approach for fabricating a touchless UV sensor can be summarized as follows. Once the sensor requirements such as flexibility, sensitivity, detection range, response time, and form factors are finalised, the UV light detection technology can be determined. Photodiodes (Liu et al., 2021), phototransistors (Xie C. et al., 2020), and UV-sensitive films (Kutepov et al., 2019) are examples. UV-sensitive films can be a good option because they can conform to different shapes in this regard. To retain conformity, soft sensors can make use of flexible substrates such as silicone or elastomer. However, the fabrication process must ensure that the sensor design allows in UV light exposure to the sensitive area of the sensor. Therefore UV LEDs can be a good source of UV light. Although the position of the light source provides optimal illumination for the UV-sensitive area, it should also retain the conformability of the sensor. The remaining procedures, such as circuity, signal processing, calibration, and testing, follow the same procedure as the other soft sensors in the field.

In summary, UV sensing is still in the emerging stage and has fewer applications in soft robotics than UV actuation. UV and IR-based technologies share common constraints in the development of sensors in some aspects such as slow work in progress, higher safety requirements, reliance on the photoelectric effect etc. Furthermore, stretchable materials with UV sensitivity are scarce at present. However, certain aspects common to sensors can easily be adopted for soft touchless UV sensors. UV sensors achieved very low sensor sophistication due to the above reasons, including safety constraints in handling UV light.

### 2.6 Acoustic sensing

Acoustic sensing has been deployed in soft robots, primarily for voice recognition and navigation. Acoustic sensors are less accurate and are susceptible to environmental noise. Despite the advances in soft acoustic sensors, considerable success has been achieved.

Hydrogel-based acoustic sensors are highly sensitive supercapacitive stress sensors that can electrically measure sound pressure. Gao et al. (2016) presents an acoustic sensor of 9 mm^2^ made by integrating an easily deformable network of metal nanoparticles in a hydrogel matrix for use as a cavity-free microphone that responds to underwater acoustic waves over a wide range of frequencies (20–3,000 Hz). Because the acoustic impedance of a hydrogel is almost perfectly matched with that of water, hydrogels are frequently used in soft robotic applications underwater.

A highly conformal device-skin contact with soft acoustic sensors and loudspeakers–is widely deployed in immersive AR/VR applications in Wang K. et al. (2021). Metallic nanowires have proven to be promising materials for conformal and wearable acoustic sensors (Kang et al., 2018; Chen W. et al., 2020; Gong et al., 2020) and loudspeakers (Bobinger et al., 2018) owing to their high aspect ratios and intrinsically flexible nature. According to these studies, the mesh-like morphology and high conductivity of nanowire-based thin films allow excellent transparency and conductivity with nanoscale thicknesses. Moreover, the intrinsic stretchability of nanowire materials, such as vertically aligned gold nanowires, offers very high mechanical and electrical properties for acoustic sensors, even under large mechanical deformation. The nanowire thin film exhibited a high sensitivity with very high-frequency discrimination up to around 3,000 Hz, which covers the most commonly used frequencies for human voice recognition. In addition, ultrasensitive acoustic sensors exhibit high-frequency selectivity of approximately 319–1951 Hz, which is similar to the function of the human cochlea (Gong et al., 2020).

A microphone is embedded into the air chamber of the actuator, where any contact with the environment induces sound (vibration) in the actuator (Zöller et al., 2018). This could not be considered soft sensing since the microphone is a MEMS (Micro-electro-mechanical systems) made of rigid materials. However, this sensor could be used in soft membranes for the purpose of touchless sensing as long as it does not limit physical compliance of the material. In contrast, a soft resistive artificial basilar membrane (ABM) or a microphone is implemented in Gong et al. (2020). Several resistive nanomaterials including carbon nanotubes, Yamada et al. (2011); Liu et al. (2015) nanowires (Kang et al., 2018), nanoparticles (Zhang et al., 2017), nanosheets (Lim et al., 2016), and graphene (Tao et al., 2017) have been used to sense acoustic vibrational forces, indicating their potential for voice recognition applications (Ding et al., 2019). In addition, acoustic cameras recently developed for industrial purposes combine many soft membranes to improve their functionality. These are arrays of microphones used to localise sound sources and visualize their properties in a given environment (Busset et al., 2015).

To summarise, soft acoustic sensors have primarily been deployed in applications such as auditory membranes which include microphones and loudspeakers. Fabricating these sensors involves a diverse range of materials, including hydrogels and carbon nanowires, and various techniques, such as MEMS. A larger variation between the working principles of sensors can be observed in acoustic sensors than in other sensing technologies. The moderate speed, being less harmful to living organisms, and diverse applications of sonar, ultrasound technologies could be identified as the main reasons for this. Ultrasonic sensors achieved low sensor sophistication due to the above reasons, including sound waves being often distracted by the disturbances.

## 3 Multimodal sensor fusion

### 3.1 Virtual sensors

A soft or virtual sensor is a software or a part of an algorithm that can be created by combining signals obtained by one or more physical sensors and processing further to get more information in addition to direct measurements from standalone sensors. Here “soft” refers to virtual information which is different from the same word being used to refer to “conformity” in soft robotics. Virtual sensing is popular in applications where the physical implementation of sensors, and other equipment is not economically viable and there are other resource constraints. Furthermore, this concept supports the reusability of resources, and hence, has become popular in sustainable sensory solutions. This requires middleware to communicate with the physical components of an application. For instance, the virtual sensor running on a sensor network on a crane in Kabadayi et al. (2006) allows an application on a person’s mobile to sense a danger circle for nearby cranes. This predicts that the areas are unsafe to walk and are centered at the base of the crane, which expands or contracts as the position of the crane, crane arms, boom, etc., ensuring the safety of workers. They have been widely used for the control, monitoring, and optimisation of industrial processes (Jiang et al., 2020). Although not popular in soft robots, this can be a good alternative where using multiple sensors will affect the flexibility of the robot. Although existing technologies do not permit it, the concept of soft sensing has the potential to integrate inputs from multiple embedded sensors and processes in a soft robot.

Millions of sensors currently contribute to the information systems. Hence sensors attached to cyber-physical systems in the real world can be used in soft robots, especially in cases where space becomes a constraint to implant adequate sensors (Martin et al., 2021). The integration of the physical sensor output into information systems comes has several limitations. These include the high cost of equipping assets with sensors, presence of noisy sensor signals or signal interference, potential loss of sensor accuracy over time, and technical infeasibility of the sensor used in certain spatial or environmental conditions.

Nevertheless, software-based virtual sensors provide an additional layer of abstraction that relies on digital representations of sensor hardware. These virtual sensors generate signals by aggregating inputs from physical sensors, thereby potentially overcoming the aforementioned limitations. They offer a wide range of advantages such as lower operating costs, increased reliability, enhanced agility, reduced space consumption and the ability to indirectly measure physically non-measurable properties (Liu et al., 2009) Moreover, virtual sensors enable the broader availability of low-level physical sensor information for application in cyber-physical systems.

Virtual sensors promote collaboration among various sources of information at multiple levels. At the sensor level, they facilitate improvements in the accuracy of individual sensors. At the asset level, they enable replacement or substitution of individual sensors. Furthermore, at the organizational level, virtual sensors empower different service providers to offer services based on the same sensor hardware. As a result, while physical sensors typically cater to specific and isolated applications, virtual sensors have become the primary source of physical world data for generalized and interconnected cyber-physical systems Martin et al. (2021). If the concept of virtual sensors is properly deployed in soft robots, these sensors can achieve high sensor sophistication due to the maximum efficiency over limited resources and easy implementation.

### 3.2 Multimodal sensors

Some sensors use more than one sensing technique for perception. These sensors were analyzed using multimodal sensors.

A textile-only capacitive, pressure, and non-contact bimodal fabric-only capacitive sensor with a high sensitivity and ultralight detection was implemented in Ye et al. (2022). Graphene nanoplatelet-decorated multidimensional honeycomb fabric and nickel-plated woven fabric served as the dielectric layer and electrode, respectively. This is a bimodal sensor with a pressure-sensing sensitivity of 0.38 kPa^−1^, an ultralow detection limit (1.23 Pa), noncontact detection performance with a detection distance of 15 cm, and a maximum relative capacitance change of 10%. Such sensors can successfully detect subtle human motion such as during finger bending and swallowing saliva. However, many challenges remain in developing a fully integrated textile sensor array with adequate flexibility, high sensitivity, multisensing capabilities, and ultralight detection. A flexible bimodal smart skin (FBSS) based on triboelectric nanogenerators and liquid metal sensing that can perform simultaneous tactile and touchless sensing and distinguish these two modes in real-time (Liu W. et al., 2022) is a multimodal teachable soft interface that reacts to both touch and touchless stimuli. The soft robotic skin made with a laser-patterned kirigami structure of a sensor network was applied on a soft gripper (Ham et al., 2022). This includes both proximity and temperature sensing, which uses the changes in capacitance and resistance, respectively. These examples demonstrate that stretchable electronics play a vital role in the manufacture of soft-touchless sensors.

Graphene-based sensors are smart multifunctional sensors (Jin et al., 2020). These sensors have been used as textcolorredUV/fluorescence (Cao et al., 2017) and gas/electrochemical sensors (Vasseghian et al., 2021) as required. Soft robots are fabricated by incorporating magnetized NdFeB patterns and have both tactile and touchless sensing modalities, including color changes upon UV light and external magnetic fields (Dong et al., 2022). A magnetosensitive skin that extracts information from its surroundings using magnetic tags was presented in Cañón Bermúdez et al. (2018). A set of spin valve sensors is arranged considering their exchange bias direction in two Wheatstone bridges, each containing four spin valve sensors, to realize the 2D magnetic field sensor. These sensors were fabricated on ultrathin polyimide foils that can be applied to human skin. Skin friendliness is another sort-after characteristic of soft touchless sensors, which broadens their applications. A promising application of mixed modality sensing in soft robotics is soft skins and (Shih et al., 2020) provides a range of examples in this regard. To interpret sensor information, machine learning techniques have been utilized to decode the physical deformation of the mechanical structure (Chin et al., 2020).

**FIGURE 2:**
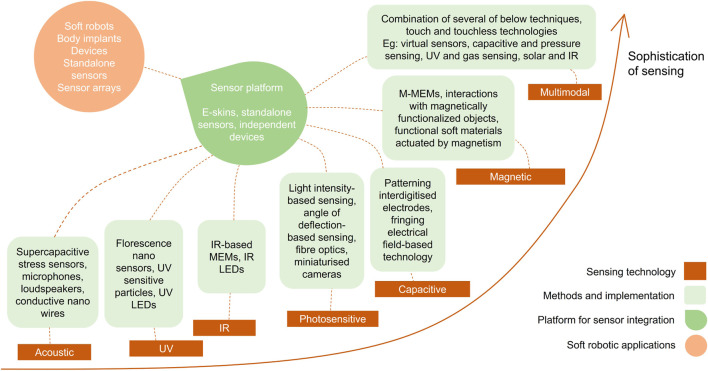
The general observation of sensing sophistication considering existing soft touchless sensing technologies.

To summarize, soft robotics currently has a range of touchless sensing technologies. However, the production of accurate sensors is hindered by the challenge of identifying conformable materials and fabrication methods. Compared with rigid sensors in the market, soft sensors generally have lower sensitivity and accuracy. Sensing sophistication is influenced by factors such as sensing technology, fabrication techniques, materials, and applications. Sensitivity and accuracy are particularly important, although soft sensors are yet to achieve significant progress in these areas. It is worth considering that some soft sensing modalities possess higher sophistication over others and multimodal sensing typically achieves higher sophistication in sensing since multiple sensing modalities have been combined. By combining different sensing techniques, multimodal sensing can enhance one’s ability to perceive and understand the surrounding environment. This can be particularly useful in soft robotics applications, where the integration of multiple sensory inputs can provide a more comprehensive and reliable understanding of the environment with limited locomotion. A general observation of sensing sophistication based on the progress in sensing technology in this field is shown in Figure 2. It can be seen that some sensing technologies intersect with each other in terms of sensor sophistication. In this regard, sophistication describes the data availability of a sensor, multi-sensing capabilities, networking with other components, adaptability, energy efficiency, and integration of many other aspects. In this study, we considered the advancement of sensing technologies, the resolution and range of sensors and other technical aspects of sensors, performance of sensors, availability of sensors to be used in real world applications, progress of fabrication techniques and materials, and if they are used in any soft robotic platform at present, to determine sensor sophistication. This categorisation might change depending on the advancement of materials and fabrication techniques in the future. Multimodal sensors can achieve high sophistication in sensing once advanced sensing techniques and principles of operation have been combined, with limitations in sensor fusion technologies and data accessibility.

### 3.3 Multifunctional sensing architectures

Multifunctionality can be useful for sensors to perform multiple tasks, including sensing. Reduced system complexity, conformability, space and cost efficiency are some aspects of touchless sensors in this regard. Although touchless sensing is important, as soft sensors are embedded in highly flexible interfaces, having tactile information between the sensor and the environment can help the sensor’s survival. This will further help realise the mechanical properties of the associated materials. The embedded soft sensor exterior prototype in Kadowaki et al. (2009) detects 3D deformation of the sensor. The sensor is made by molding soft urethane foam with LEDs and light-receiving devices where multi-axis deformation can be detected by the voltage changes of each light-receiving devices. It is interesting that these are small scale (7*6 mm) and are capable of detecting loads until 30 kg in weight. Most of the mechanical constraints associated with soft sensors have been overcome by this setup. Reda et al. (2011) is an example of soft sensor allocations used on data from embedded, rigid thermal sensors, to track and manage the hotspots during the runtime of processors.

Escobedo et al. (2020) discusses an energy-generating eSkin with intrinsic tactile sensing without using touch sensors. Instead comprises a distributed array of miniaturized solar cells and IR light emitting diodes (IR LEDs) on the soft elastomeric substrate. Shadow sensing could sense multiple parameters including proximity, object location, and edge detection using this technique. Using mixed physical modalities in a sensor as in Majidi (2020) can solve the problems of space, sensing capability, etc. in soft robots.

Sensors can be manufactured using a variety of methods, depending on the type of sensor and the desired properties. Some common manufacturing methods apply to soft sensors as well. For instance, microfabrication, 3d printing, hot embossing, chemical deposition, and mechanical casting. Manufacturing techniques might affect the required resolution, accuracy, and mechanical behaviors of the sensor.

### 3.4 Embodied energy, sensor morphology and soft touchless sensors

Sensing and embodied energy are significant concepts in the field of robotics, each playing a unique role in the design, operation, and sustainability of robotic systems. Touchless sensing has some added benefits in this regard as it enhances safety by reducing the need for physical interaction, increases precision and efficiency in sensing, and expands the range of environments where robots can operate. As sensors add up to the energy consumed in the production, transportation, and disposal of a robot, energy efficiency in touchless sensors plays a major role in the sustainability of robotic systems. We identify balancing the embodied energy with the operational efficiency of robots and sourcing materials and components with lower environmental impact as two main challenges towards this end. Hence integrating soft robotics, touchless sensing and principles of embodied energy can be a promising direction to increase soft robotic sensing as well as harvest energy efficiently from the environment.

Embedded multifunctionality, flexibility, structural compliance, and energy efficient sensing capability provide a new paradigm for soft robots (Aubin et al., 2022). The flow sensor in Ishida et al. (2019) is an example for this. This sensor identifies significant changes in flow, detecting an increase in flow within the hydrodynamic environment and enables the extraction of underwater hydrodynamic information, such as the speed of the flow. Furthermore, most current robotic systems still use isolated power, actuation, sensory and control blocks, etc. Machine autonomy in such systems could be improved by developing multifunctional embodied energy systems with the help of soft robotics and touchless sensing. Concepts related to harvesting, storage, application, and recovery of energy throughout the touchless sensing-enabled soft robotic systems are yet to be improved to solve real-world design challenges. Materials play a prominent role in the success of these combined technologies (Chen Z. et al., 2020). Hence touchless sensing for soft robots can be considered as a requirement of adaptation of sensor morphology. This further requires the sensor morphology adaptation from a bioinspired perspective that includes the design/planning of the morphology, self-assembly using the necessary source materials, as well as the sensing and evaluation of performances based on task–environment interactions (Iida and Nurzaman, 2016).

## 4 Challenges and remarks

The main difference between soft- and rigid-bodied robots centres that they seamlessly combine the actuation, sensing, motion transmission, and conversion mechanism elements, electronics, and power sources into a continuum body that ideally holds the properties of morphological computation and programmable compliance (i.e., softness) (Alici, 2018). Soft smart materials with programmable mechanical, electrical, and rheological properties, and conformability for additive manufacturing based on 3D printing are essential to realise soft robots (Alici, 2018). Although soft robots cannot replace conventional robots, they are ideal for some applications, especially medical applications. However, the sensing requirement of soft robots keeps increasing as the demand increases. Hence, improving the sensing capabilities of soft robots is essential because of their widespread use textcolorred. Close collaboration between soft robotics and material science can leverage this task exponentially. This was justified by the touchless sensors reviewed in this study.

Another aspect of soft-bodied robots is that they involve infinite DOFs. Thus, it is challenging to develop kinematics for modelling them. Hence advances in integrated or distributed touchless sensing modalities are conducive to the progress of soft robot control strategies. The sensitivity of a soft touchless sensor refers to its ability to detect small changes in the physical environment. Different types of soft touchless sensors have varying levels of sensitivity. Optical sensors, such as photodiodes and phototransistors, are generally considered to be among the most sensitive soft touchless sensors. They can detect changes in light intensity with high precision and a wide dynamic range. However, their sensitivity can be affected by the environment, such as ambient light, and they may require specialized filters or lenses to improve their performance. Acoustic sensors such as piezoelectric sensors and microphones are highly sensitive and can detect changes in sound pressure levels with high precision. They can be used to sense vibrations, pressure changes, and sound waves. Electromagnetic sensors, such as Hall effect sensors, are highly sensitive to magnetic fields and can detect changes in magnetic field strength with high precision. They can be used for sensing magnetic fields or for detecting the presence of magnetic materials.

### 4.1 Applications

Soft sensors have been textcolorred effectively used as implants in the human body. An average human comprises a 14% skeleton (Shephard and Shephard, 1991) and the remainder comprises most of the soft tissue. Hence, soft components, including soft sensors, are considered as implants to treat the human body during surgery. These have become a part of minimally invasive surgery (Runciman et al., 2019). Hence, soft sensors have potentially strong applications and demand despite the challenges associated with their advancement.

To accommodate complex robot behavior of soft mechanisms, three-dimensional integration of both sensing and actuation has challenges in both technology and fabrication. There are several requirements for integrated sensing elements in soft sensors. First, they must be sufficiently compliant to restricting or extensively modifying the properties of soft robots. Second, they must be resilient and extensible to prevent failure over many motion cycles. Third, they cannot possess features that act as stress concentrators and hence cause damage (Polygerinos et al., 2017). The evolution of e-skins (Hammock et al., 2013) and epidermal electronics replicating the human skin (Webb et al., 2013) testifieshis.

### 4.2 Design

As a whole, the sensitivity of a soft touchless sensor depends on the type of sensor and the application. Optical sensors are generally considered to be among the most sensitive, but acoustic and electromagnetic sensors can also provide high sensitivity in certain applications. The reason for this is that light and electromagnetic radiation are faster in transmitting from one medium to another and acoustic sensor technology has been extensively studied during the past decade or so. Other than that, the concerns common for all sensors in general, for instance, sensitivity, flexibility, durability, signal-to-noise ratio, power consumption and cost, etc. apply to soft touchless sensors as well. Soft sensors typically have lower sensitivity compared to traditional rigid sensors, which can make it difficult to detect small changes such as pressure or movement. Soft sensors need to be flexible and conformable to be able to conform to the surface they are measuring. In terms of durability, soft sensors are often made of flexible materials, which can be prone to wear and tear over time. Therefore solutions are required to make soft materials durable (Li and Guo, 2019). Furthermore, soft sensors may require a lot of power to operate, which can be a challenge when designing battery-powered devices. The cost of developing and manufacturing soft sensors can be high, which can make them less accessible to some applications.

### 4.3 Fabrication

There are various ways to fabricate touchless sensors in soft robotics. In optical sensing, one of the popular methods is to embed optical fibers into soft materials (Fu et al., 2018), such as silicone or rubber, which can then be used to detect changes in light transmission or reflection. Similarly, soft capacitive sensors can be fabricated by depositing conductive materials, such as metal or carbon nanotubes (Yamada et al., 2011), onto soft materials. In addition, some sensors can be fabricated by incorporating conductive particles, ferromagnetic or superconductive materials, or nanocomposites into soft materials. The choice of fabrication method will depend on the specific application and the desired level of sensitivity and accuracy. For instance, laser ablation strategy and plastic cutting have been used to stretch an expandable multi-modal sensor network around a soft skin (Ham et al., 2022). MEMS are another technology supporting soft structures that not only simplifies sensor architecture for fabrication but also avoids interference from non-relevant objects (Ge et al., 2019). Various manufacturing methods such as microfabrication, inkjet printing, hot embossing, CVD, deposition, assembly, and testing are used to produce soft touchless sensors.

3D printing is a popular method for fabricating soft robotic systems due to its ability to produce complex geometries and customizable designs (Preechayasomboon and Rombokas, 2020). It can also be used to create both the soft and rigid components of a soft robotic device. The same technique is adopted in embedding sensors in soft robots as well (Valentine et al., 2017; Zhao et al., 2021). Lost filament, and stereo lithography can be cited as examples for this. Additionally, 3D printing can also be used to create molds for casting soft materials, such as silicone or rubber, which can be used to create complex, flexible sensors. Nanotechnology, especially nanosheet thin films can be a good match to be incorporated with flexible materials in the future (Szendrei et al., 2015). The availability of printing and casting technologies define the majority of the characteristics of a soft sensor. For instance, printing and casting technologies help create sensors by enabling miniaturization, customization, rapid prototyping, the creation of complex structures, and the integration of multiple sensing modalities into one device.

Glass–coated microwires are one of the families of magnetic microwires that has a higher optimization of magnetic softness, giant magnetoimpedance (GMI) effect, and domain wall dynamics (Zhukov et al., 2020). They achieved a high magnetic field resolution-hence ideal for sensors. Soft magnetic micro and nanowires came into play as the requirement of wires with more reduced dimensions in magnetoelastic applications Vázquez (2001). Due to the size of nano and microscale sensors, they have become extremely easy to embed into soft polymers without hindering their flexibility.

### 4.4 Materials

Touchless technologies make use of different materials for sensing. These materials include soft alloys (Zhukov, 2017), air (Wang et al., 2022a), light (Wu et al., 2021), capacitance (Ham et al., 2022), magnetism (Dong et al., 2022), IR (Muthuviswadharani et al., 2016), acoustic (Kang et al., 2018) and virtual technologies (Mattera et al., 2018). Improved fabrication techniques, resolution, and range in recent years largely expanded the application of sensors made of soft materials or noncontact media. For instance, nowadays there are fibres at different scales, such as micro and nano, which possess different conformability. The reason for this is their composition and the nature of fiber assemblies (Zeng et al., 2014). Therefore similar materials could be used in fabricating soft touchless sensors. Evenso, fabrication techniques and lack of materials with the required properties are the most prominent drawbacks to the development of touchless sensors. Space limitations, power, and control have always been barriers to building soft robots and making use of their embodied energy. Hence rigid materials play a remarkable role in making soft robots fully equipped and functional as rigid robots Aubin et al. (2022). However, the requirement of touchless sensing cannot be neglected with the increase of soft robotic applications in the real world Woodington et al. (2021). One major challenge in this regard is the different fabrication techniques involved with each sensing technology.

A summary of different sensing modalities and their recent progress and challenges related to these aspects are highlighted in Tables 1, 2.

**TABLE 1:** Summary of typical touchless sensors and the corresponding soft robotic case studies.

Sensing techniques	Materials	Measurements	Fabrication
IR	Silicone Meng et al. (2020); Muthuviswadharani et al. (2016)	distance Meng et al. (2020)	Hybrid Deposition Manufacturing (HDM) Meng et al. (2020)
	Photointerrupter Ogata et al. (2013)	photo reflectivity Ogata et al. (2013)	MEMS Muthuviswadharani et al. (2016)
		temperature Yamaguchi et al. (2019)	assembly Ogata et al. (2013)
		proximity	Muthuviswadharani et al. (2016)
Acoustic	Metal nanoparticles (MNPs) Gao et al. (2016)	acoustic waves Gao et al. (2016); Wang et al. (2021b)	MNP network Gao et al. (2016)
	AgNPs Bobinger et al. (2018)		deposition + spray + photonic sintering Bobinger et al. (2018)
	Gold nano-wire (V-AuNW) films Gong et al. (2020)		multi-layer array Wang et al. (2021b)
	AgNW Kang et al. (2018)		multi-layer + dissolvation Gong et al. (2020)
	Metallic nanowires Wang et al. (2021b)		multi-layer array Kang et al. (2018)
Magnetic	NdFeB microparticles Dong et al. (2022)	temperature, UV light, pH	multilayer film and adhesion Dong et al. (2022)
	PDMS + Magnetic film + GMR sensor+	circuit, position	
	Polyimide Ge et al. (2019)	oil sensing Dong et al. (2022)	
	PDMS–nanorod composite Stottlemire et al. (2021)	magnetic field strength Stottlemire et al. (2021)	extrusion-based 3D printing Stottlemire et al. (2021)
	FeNi alloy + silver nanowire-coated		
	PDMS Cai et al. (2018)	proximity, pressure Ge et al. (2019)	m-MEMS Ge et al. (2019)
		curature Cai et al. (2018)	Moulding and embedment Cai et al. (2018)
Ultraviolet	Graphene quantum dots (GQDs)+	fluorescence Cao et al. (2017)	hydrothermal method Cao et al. (2017)
	Graphene oxide (GO) Cao et al. (2017)		
	NdFeB microparticles Dong et al. (2022)	Temperature, UV light, pH, circuit	multilayer film and adhesion Dong et al. (2022)
Photosensitive	Soft substrate Dobrzynski et al. (2011)	position, oil sensing Dong et al. (2022)	surface energy-induced
	Stretchable phototransistors	Angles, deflection Liu et al. (2022a)	self-assembly methodology Liu et al. (2022a)
	Hybrid polymer semiconductors	proximity Guo et al. (2019a)	m-MEMS, multi-layer application Guo et al. (2019a)
	Hydrogel Liu et al. (2022a)		
	Biodegradable materials, hydrogel Guo et al. (2019a)		
Capacitive	Carbon nanotubes	proximity Wang et al. (2023)	sacrificial template method Wang et al. (2023)
	Polydimethylsiloxane (PDMS)	touch	
	Polyethylene terephthalate (PET)		
	Polyvinyl alcohol (PVA), Ecoflex		
	Other conductive materials		
	with polymer substrates		
Virtual	none	any based on other sensory inputs	None
Multimodal	A combination of a	proximity, temperature, pressure	one or a few of above techniques
	few above depending on	motion, luminescence	
	the sensing techniques used	capacitance and resistance	
		fluorescence Ye et al. (2022)	
		touch Liu et al. (2022b)	
		magnetism Cañón Bermúdez et al. (2018)	

**TABLE 2:** Summary of typical touchless sensors and the corresponding soft robotic case studies-continued.

Sensing techniques	Remarks	Applications
IR	soft palm protects on-board IR sensor Meng et al. (2020)	soft robotic gripper
	Fused with IMU, gyro Muthuviswadharani et al. (2016)	autonomous vehicle
	human body as a part of input Ogata et al. (2013)	sensorized arm band
	multiple sensor integration Yamaguchi et al. (2019), low sensor sophistication	soft robotic hand
Acoustic	underwater acoustic signals 20 Hz to 3 kHz Gao et al. (2016)	hydrogel microphone, may enable imperceptible
	maximum return loss and radiation of 27 dB and 95% Bobinger et al. (2018)	coplanar/flexible antenna
	health monitoring during future	AR/VR
	AR/VR practices Wang et al. (2021b), high-frequency	artificial basilar membrane
	selectivity in the range of 319–1951 Hz	skin-attacheable loudspeaker
	and high sensitivity of 0.48–4.26 Pa1 Gong et al. (2020)	
	addressed the NM-based conformal electronics	
	required foracoustic device platforms Kang et al. (2018), low sensor sophistication	
Magnetic	modular soft material systems Dong et al. (2022), Images from microscopes	multifunctional magnetic
	and magnetic hysteresis loops captured by superconducting	soft robots
	quantum interference device can be used for characterisation Guo et al. (2019a)	curature detection
	sensing deformation and altering stiffness in	remote sensing
	the presence of an external magnetic field Stottlemire et al. (2021)	and remote actuation
	simultaneously tactile and touchless sensing Ge et al. (2019), Moderate sensor sophistication	e-skin
Ultraviolet	GQDs-GO shows a high selectivity for Cyt c detection, exhibits	cytochrome detection
	favorable intracellular imaging in A549 cells Cao et al. (2017)	multifunctional
	modular soft material systems Dong et al. (2022), Very low sensor sophistication	magnetic soft robots
Photosensitive/Optical	stretchable, soft	wearables, implants
	soft or rigid, stretchable/unstretchable, biocompatible	gap detection
	biodegradable materials	
	microfabrication is a possible future trend, Moderate sensor sophistication	
Capacitive	fabrication can be scalable Wang et al. (2023), Moderate sensor sophistication	wearables
Virtual	intangible, Possibility of high sensor sophistication based on data sources	all the sensory platforms
Multimodal	Usually sensor arrays present, Higher sensor sophistication	wearables, robots
		biomedical devices, vehicles

**FIGURE 3:**
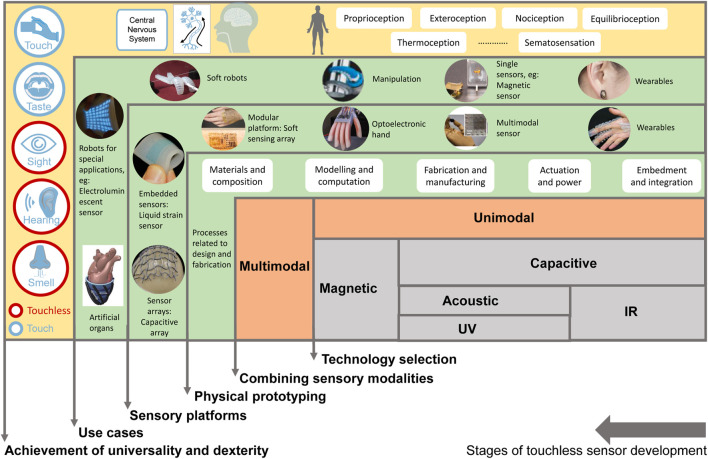
Trends in the development of sensing technologies and pathway towards achieving human-level dexterity and sensory universality. Starting from right to left, different sensing techniques which leads to multimodal sensing, fabrication of physical prototypes, sensory platforms where these sensors are integrated, real world applications of these platforms are illustrated. Finally, with the advancement of all the above aspects, aims for achieving universality and dexterity. These aspects are shown as the stages of development of sensors. Sensing techniques with low sophistication are shown on the right and the sophistication of sensing increases as it goes from right to left. IR, Acoustic, UV sensing techniques achieved low sensor sophistication and capacitive and magnetic sensing which achieved a moderate sophistication. Unimodal sensors in general can be considered as less sophisticated in performance. Hence the highest sophistication in sensing was achieved by multimodal sensors. The range of sensory platforms comprises various innovative technologies, such as the sensory glove (Kang et al., 2019), optoelectronic hand (Zhao et al., 2016), multimodal sensory chip (Yang et al., 2022), modular soft sensing array (Kim et al., 2011), embedded liquid strain sensor (Chossat et al., 2013), and capacitive sensory array integrated onto a universal gripper (Loh et al., 2021). These platforms find diverse application scenarios, including wearables (Heo et al., 2018), magnetic electrostatic sensors (Liang et al., 2021), compliant manipulation inspired by octopus movements (Xie Z. et al., 2020), soft pneumatic locomotion (Tolley et al., 2014), electroluminescent sensing (Larson et al., 2016), and artificial organs (Roche et al., 2017).

In summary, in order to create high-density and multifunctional sensors for soft robots, innovations in various aspects are required. This will require a close collaboration between roboticists, physicists, materials scientists, and many more to develop high-performance stretchable materials with required mechanical and electrical properties. It will also be necessary to explore different sensing modalities and integration architectures. Finally, when designing sensory systems, hardware and algorithms for data processing should be taken into consideration, and their performance should be evaluated on a wide range of practical robotic applications. This is illustrated in Figure 3. The figure elaborates on the process of evolution of a soft (touchless) sensor with respect to technologies, sensing modalities, and fabrication until it receives a high level of dexterity which is often compared to the human level. We show several important stages of sensor development here. These stages are categorised according to the current challenges and research focuses. Starting from the bottom right, depending on the application and the existing boundaries, the first step toward the development of a touchless sensor will be technology selection. There can be more technologies related to touchless sensing in the future, in addition to the ones highlighted in this review. The next stage is combining sensory modalities: unimodal and multimodal, to combine multiple sensing technologies to improve the sensing performance. Then physical prototyping can be started and this includes the process of modelling, design, fabrication, and control of the sensor. These sensors can be integrated into sensory platforms depending on a sensor’s use case. Major use cases of soft touchless sensors are standalone sensors, wearables, robot manipulators, and inner-body applications. While this is the process of the development of a soft touchless sensor, the ultimate goal of the process of soft touchless sensing is the achievement of universality and dexterity. However, to achieve a high level of dexterity as in humans, it is important to combine tactile and touchless sensing and process it effectively. The nervous system is a good example of the instinctive handling of multiple sources of sensory information, by incorporating sensory inputs, motor outputs, communication between organs, internal stability, learning, and memory. Integrating soft robotics with touchless sensing technologies is seen to be an effective strategy for enhancing the dexterity of robotic systems.

## 5 Conclusion

The introduction of soft touchless sensing into human-robot environments opens up avenues for exploring novel frontiers within the realm of human-robot interactions. In this review, we provide an overview of soft robotic systems embodied with touchless sensing. From the past to the present, the focus of researchers in soft robotics shifted from actuation to sensing and then multiple aspects together: sensor-actuation-embodiment-coordination-outreach. In this review, we identified the limitations of existing systems and suggested possible future improvements and future directions. This review provides principles and approaches for the further development of touchless sensing mechanisms for soft robots. In summary, the capability of existing touchless sensing technologies for soft robots is far lower than that for rigid robots. Simultaneously, soft robots face more challenges in terms of structure, composition, actuation, sensing, and perception. Furthermore, the literature related to soft robots with touchless sensing is relatively scarce, even though there is great potential for development in this aspect. Despite these challenges, research in this area continues to make advancements and improve the performance and capabilities of soft touchless sensors.
